# Sex-specific phenotypical, functional and metabolic profiles of human term placenta macrophages

**DOI:** 10.1186/s13293-024-00652-w

**Published:** 2024-10-17

**Authors:** Daniel E. Paparini, Esteban Grasso, Franco Aguilera, M. Agustina Arslanian, Victoria Lella, Brenda Lara, Ana Schafir, Soledad Gori, Fátima Merech, Vanesa Hauk, Claudio Schuster, Marcelo Martí, Cesar Meller, Rosanna Ramhorst, Daiana Vota, Claudia Pérez Leirós

**Affiliations:** 1 Immunopharmacology Laboratory, Instituto de Química Biológica de la Facultad de Ciencias Exactas y Naturales (IQUIBICEN), Universidad de Buenos Aires-Consejo Nacional de Investigaciones Científicas y Técnicas (CONICET), Buenos Aires, Argentina; 2 Bioinformatic Laboratory, Instituto de Química Biológica de la Facultad de Ciencias Exactas y Naturales (IQUIBICEN), Universidad de Buenos Aires-Consejo Nacional de Investigaciones Científicas y Técnicas (CONICET), Buenos Aires, Argentina; 3 Obstetric Service, Hospital Italiano, Buenos Aires, Argentina

**Keywords:** Placental-macrophages, Metabolism, Sex-associated differences

## Abstract

**Background:**

Placental macrophages, Hofbauer cells (HBC) are the only fetal immune cell population within the stroma of healthy placenta along pregnancy. They are central players in maintaining immune tolerance during pregnancy. Immunometabolism emerged a few years ago as a new field that integrates cellular metabolism with immune responses, however, the immunometabolism of HBC has not been explored yet. Here we studied the sex-specific differences in the phenotypic, functional and immunometabolic profile of HBC.

**Methods:**

HBC were isolated from human term placentas (*N* = 31, 16 from male and 15 female neonates). Ex vivo assays were carried out to assess active metabolic and endoplasmic reticulum stress pathways by flow cytometry, confocal microscopy, gene expression and in silico approaches.

**Results:**

HBC from female placentas displayed a stronger M2 phenotype accompanied by high rates of efferocytosis majorly sustained on lipid metabolism. On the other hand, male HBC expressed a weaker M2 phenotype with higher glycolytic metabolism. LPS stimulation reinforced the glycolytic metabolism in male but not in female HBC. Physiological endoplasmic reticulum stress activates IRE-1 differently, since its pharmacological inhibition increased lipid mobilization, accumulation and efferocytosis only in female HBC. Moreover, differential sex-associated pathways accompanying the phenotypic and functional profiles of HBC appeared related to the placental villi environment.

**Conclusions:**

These results support sex-associated effects on the immunometabolism of the HBC and adds another layer of complexity to the intricate maternal-fetal immune interaction.

**Graphical abstract:**

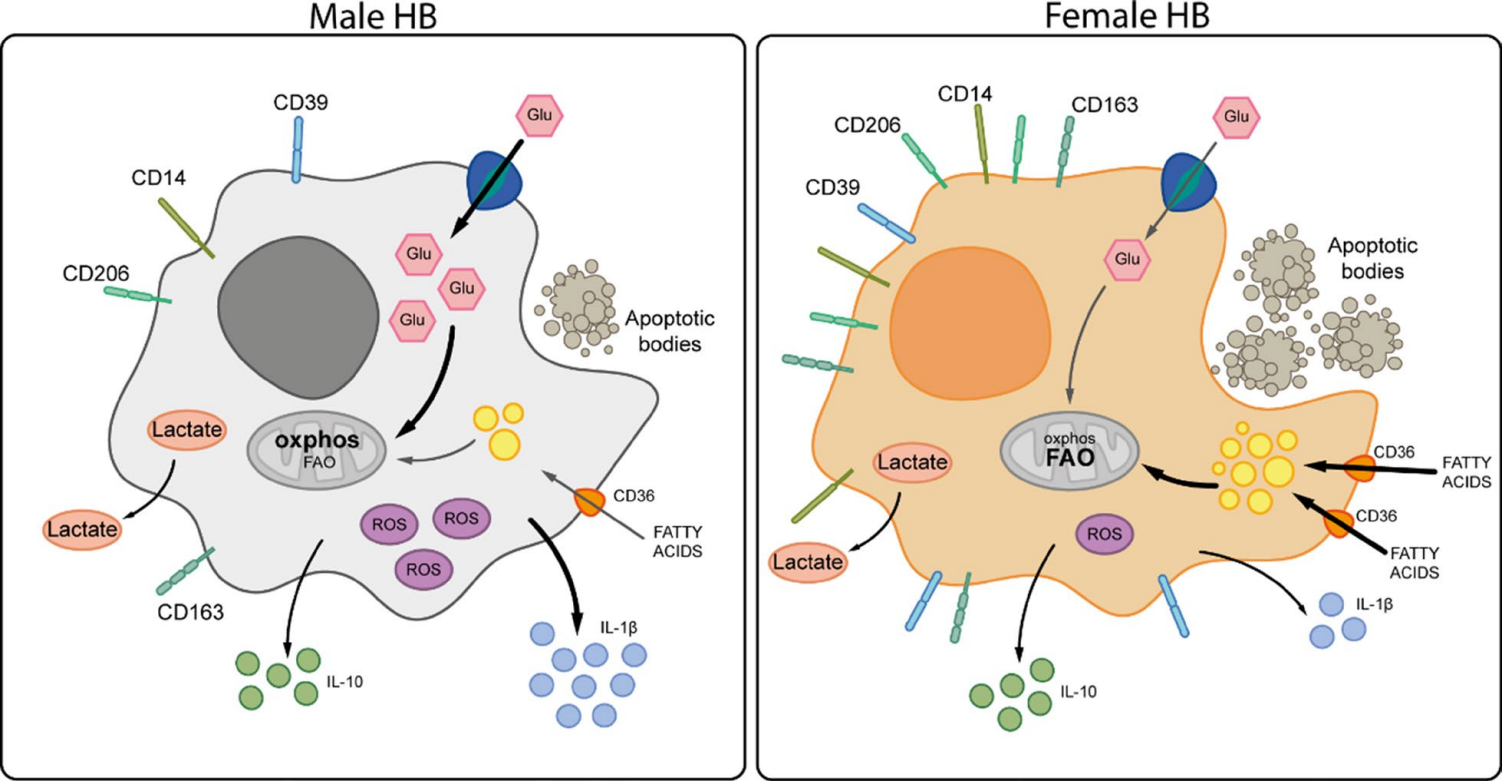

**Supplementary Information:**

The online version contains supplementary material available at 10.1186/s13293-024-00652-w.

## Abstract (plain language summary)

Placental macrophages are the only fetal immune cell population within the stroma of healthy placenta along pregnancy and play a central role in contributing to the correct functioning of the placenta for the development of the fetus. Alterations in their metabolism lead to failures in their functions which are associated to pregnancy complications. Although, sex-specific differences were found in placental adaptation to pregnancy complications and outcomes, but the metabolism associated to their functions of placenta macrophages and whether they are associated to the sex of the placenta have not been explored so far. Here we studied human term placenta macrophages with special focus on their metabolism associated with their functions. We found out that macrophages from female placenta got energy from fatty acids whereas male macrophages used glucose. Furthermore, when female macrophages were exposed to a bacterial component, their metabolism or cellular function did not change towards one associated with a classic profile, but male macrophages did. These results might contribute to gain more insight into the immune-placental interactions at term human pregnancy and provide new clues to begin personalizing the pregnancy according to the sex of the fetus in physiological or pregnancy complications of inflammatory nature.

**Supplementary Information:**

The online version contains supplementary material available at 10.1186/s13293-024-00652-w.

## Highlights

Sex-associated diversity in placental macrophages (HBC) immunometabolism may contribute to the understanding of differential susceptibility to infections and pregnancy complications.

Under physiological stress, IRE-1α bias acts differently in male and female HBC in the endoplasmic reticulum and underlies a possible mechanism of HBC immunometabolic profiling.

**Supplementary Information:**

The online version contains supplementary material available at 10.1186/s13293-024-00652-w.

## Background

Differences in pregnancy development and outcomes associated to the fetal sex have been reported for many years [1–5]. Fetal growth is partly determined by fetal sex: at birth, male neonates tend to be larger and heavier than females regardless of placental weight [4, 6–8]. Interestingly, during gestation, male fetuses grow faster than female fetuses, what makes them more susceptible to maternal nutritional insults [3, 9, 10], exposure to chemical [11–13] and infectious agents [10, 14, 15]. Moreover, sex-specific differences were found in placental adaptation to pregnancy complications and outcomes [2, 16–18].

Hofbauer cells (HBC) are the only fetal immune cell population within the stroma of healthy placenta [19–21]. These placental cells are transcriptional and chromosomally like yolk sac macrophages [21–23]. They reside in the placenta from 18 days post-conception until term and are central players in maintaining immune tolerance during pregnancy [19, 20, 24, 25]. HBC are poorly described due to their heterogeneity. Physiologically, HBC are activated in an alternative or M2 profile that sustain tissue repair, remodeling, apoptotic cell removal (efferocytosis), angiogenesis and immunological tolerance with massive antiinflammatory mediators’ secretion [21, 25, 26]. In pregnancy complications like preeclampsia or fetal growth restriction, they switch to a predominantly classical or M1 activation and contribute to homeostasis loss at the maternal-fetal unit [24, 27], but in vitro they had showed a resistance to inflammatory cues [28]. Regarding sex-differences in the phenotypical profile of HBC in normal pregnancy, female HBC mounted a stronger and wider response against viral and bacterial ligands than male HBC [28, 29].

Immunometabolism emerged in the last decade as a novel approach that integrates cellular metabolism with immune responses [29, 30]. Macrophages activated in an M1 phenotype derive energy preferentially from glycolysis and pentose phosphate pathway (PPP) whereas proinflammatory stimuli like lipopolysaccharide (LPS), can further increase the glycolytic metabolism. On the other hand, macrophages on the alternative or M2 phenotype obtain energy majorly from oxidative phosphorylation, fatty acid oxidation and amino acid metabolism [29–32]. However, the association between macrophage phenotypic profile and their immunometabolic reprogramming can vary depending on the tissue context and the signals received from the environment. In pregnancy, recent evidence points to the role of immunometabolic reprogramming in maternal monocytes and decidual macrophages [33,^33^–^36^]. So far, the immunometabolic profiles of placenta macrophages and whether they are associated to the sex of the placenta have not been explored. Taking into account that in normal pregnancy female HBC are more likely alternative activated macrophages, we hypothesized that this phenotype was associated to a differential immunometabolic profile. Here we studied human term placenta HBC with special focus on metabolic, phenotypic and functional profiles. The potential role of endoplasmic reticulum (ER) stress signals was also analyzed.

## Materials and methods

### Placenta samples and Hofbauer cell isolation

Term placenta tissue (*N* = 31, 16 from male and 15 female placentas regarding to the sex of the singleton born) with gestational age of 39.1 ± 0.6 was collected from women undergoing programmed caesarean birth. Women participating in the study were 26 to 41 (34.5 ± 0.7) years old and all of them had normal BMI (23.6 ± 0.6). They had negative serology for HIV, HBCV, Chagas disease and syphilis and no clinical records of gestational diseases or more than one pregnancy loss. Samples from women with serious complications during pregnancy such as infections that compromised pregnancy or fetal death were excluded. Neonates with known organ malformations or congenital disorders were also excluded. Approval was obtained from Hospital Italiano of Buenos Aires Research Ethics Committee (HIBA 1681). Written informed consent was obtained from all women undergoing elective caesarean birth. Placental villi immediately beneath *decidua basalis* was dissected as described previously [37, 38] and processed for cell and tissue assays. Briefly, small decidual or villi explants of 5 × 5 × 1 mm were excised from term placenta’s cotyledons, washed with cold PBS and then cultured in 24-well polystyrene plates in 500 µl DMEM: F12 complete, with 10% FCS and 100 µg/ml penicillin/ 100 U/ml streptomycin (Life Technologies, Buenos Aires, Argentina) for 20 h. The explants were collected for RNA assays in TRizol and conserved at -80 °C until use. For Hofbauer cell isolation, 20–40 g of villi were washed thoroughly with PBS to minimize the presence of maternal cells. It was digested with collagenase II (1 mg/ml), DNAse I (0.1 mg/ml) and Trypsin-EDTA (0.25%, Gibco) for 45 min and enzymatic digestion was finished by 10% FCS and followed by Ficoll-Hypaque centrifugation, as previously reported [39, 40]. Next, to enrich the sample in placental macrophages, an extra adherent step or positive selection CD14 cell isolation kit with magnetic beads coupled antibodies (Miltenyi) was performed with a 95% of purity (90–99%). A potentil activation of HBC during purification by CD14 positive selection seems negligible, since cells were responsive to different stimuli with the mild variability expected within each sex.

### Blood samples

Blood samples were processed from pregnant women (*N* = 10, 4 male and 6 female) at the moment of cesarean birth. Neither donor was under pharmacological treatment for at least 10 days before the day of sampling. Blood was obtained by puncture of the forearm vein, and it was drawn directly into heparin containing sterile plastic tubes. Studies were approved by the Hospital Italiano de Buenos Aires Research Ethics Committee and Research Committee (HIBA 1681). Samples were collected after corresponding written informed consent was signed by the participants.

### Flow cytometry and ELISA

4 × 10^5^ HBC were cultured without or with 100 nM lipopolysaccharide (LPS) or 10 µg/ml STF-083010 (STF), inhibits IRE-1α endonuclease and mRNA splicing activity in response to endoplasmic reticulum stress but has no effect on its kinase activity, in RPMI-160 medium (RPMI) 2% FCS and after 20 h cells were recovered by Trypsin-EDTA (0.25%, Gibco) and stained for:

- *Cytokines*: with Fluorescein Isothiocyanate (FITC-), Phycoerytrin (PE-), Phycoerytrin-Cyanine7 (PECy7-) or Allophycocyanin (APC-) conjugated mAbs directed to CD14 (BioLegend Cat. No. 325617, RRID: AB_830690), CD36/FAT (fatty acid translocase. BD Biosciences Cat. No. 555454, RRID: AB_2291112), CD163/SCARI1 (scavenger receptor cysteine-rich SRCR. BioLegend. Cat. No.333605, RRID: AB_1134005), CD39/ENTPD1 (ectonucleoside triphosphate diphosphohydrolase 1. Biolegend Cat. No. 328205, RRID: AB_940423), CD206/MRC1 (mannose receptor C-type 1) and CD209/DC-SIGN (C-type lectin) (BD Pharmingen, San Diego, CA, USA. Cat. No. 17-2099-42, RRID: AB_11039758).
- *Metabolism probes*: 2-NBDG (2-Deoxy-2-[(7-nitro-2,1,3-benzoxadiazol-4-yl)amino]-D-glucose)-FITC for glucose uptake, DCFH-DA (2’,7’-Dichlorodihydrofluorescein diacetate)-FITC for total reactive oxygen species (ROS), BODIPY (4,4‐difluoro‐3a,4a‐diaza‐s‐indacene)-FL C12-FITC for long chain fatty acid uptake, BODIPY 493/ 503 for lipid droplets accumulation. All probes were obtained from Thermo Fisher Scientific.

Fifty thousand events were acquired in a FACS Aria II cytometer^®^ (Becton Dickinson)

and results were analyzed using FlowJo software (http://www.flowjo.com/).

Results were expressed as the percentage of the respective population and the quadrant was set using irrelevant isotype specific Ab and were expressed as mean fluorescence intensity (M.F.I.) or double positive cell frequencies.

Enzyme-Linked ImmunoSorbent Assay (ELISA) was performed to determine IL-1β and IL-10 levels in HBC supernatant in absence or presence of 100 nM LPS using a commercial kit (BD Biosciences), according to the manufacturer’s recommendations and as previously described [41].

### Glucose uptake

HBC supernatant was removed after 20 h in RPMI 2% FCS with or without treatments. Cells were incubated with 100 µM 2-NBDG, a fluorescent glucose analogue, and CD14-PECy7 at 37 °C, 5% CO2 in glucose‐free RPMI for 10 min. Cells were washed with cold PBS and resuspended in 2% FCS PBS (FACS solution) for flow cytometry. Results were expressed as the 2-NBDG M.F.I. in CD14 positive cells.

### Lactate secretion

Lactate concentration in cell supernatant was measured 20 h after treatments with L-Lactate kit (Wiener Lab) according to the manufacturer’s instructions.

### Long chain fatty acids uptake

After treatments, cells were stained with the specific fluorescence probe BODIPY-FL C12 as in [34]. This probe is a 12-carbon chain length saturated fatty acid linked to the fluorophore BODIPY (4,4‐difluoro‐3a,4a‐diaza‐s‐indacene), resembling an 18‐carbon fatty acid [42]. The probe was preincubated with 0.1% fatty acid free bovine serum albumin (FAF‐BSA, Sigma) for 30 min at 37 °C. Cells were washed twice with PBS and incubated with 5 µM BODIPY-FL C12 solution in serum‐free RPMI for 5 min at 37 °C, 5% CO2. Cells were washed with 0.2% BSA, resuspended in FACS solution and data was acquired as for glucose uptake assay. Results were expressed as the BodiPY FL C12 M.F.I. in CD14 positive cells.

### Lipid droplets accumulation

Cells were treated as for glucose and LCFAs uptake, then were washed twice with PBS and incubated with a 2 µM BODIPY 493/503 fluorescent probe in PBS for 15 min at 37 °C, 5% CO2. Cells were washed with cold PBS, harvested with Trypsin-EDTA (0.25%) and resuspended in FACS solution and flow cytometry was performed as described above. Results were expressed as the BodiPY 493/503 M.F.I. in CD14 positive cells.

### Efferocytosis assays

Phagocytosis of autologous apoptotic neutrophils (efferocytosis) in 3 × 10^5^ HBC CD14 positive cells cultured without or with 10 μm STF-083010 (STF) was carried out as previously reported [43, 44]. Neutrophils were obtained after the Ficoll-Hypaque gradient and subsequent Dextran purification [44, 45]. Apoptotic neutrophils were obtained after 20 h incubation in RPMI (spontaneous apoptosis) and stained with 3 µM/10^6^ cells of CFSE (Life Technologies, Buenos Aires) for 10 min in RPMI without FCS. Excess of CFSE was eliminated by serial washes with RPMI 10% FCS. The pecentage of neutrophil apoptosis was higher than 50% as determined by annexin-propidium iodide staining and flow cytometry [43, 44].

For metabolic pathways inhibition, 10 mM 2-deoxy-d-glucose (2-DG), 100 nM rotenone (ROT) or 10 μm Etomoxir were added to HBC cultures 90 min before efferocytosis assay to inhibit glucose utilization, mitochondrial electron transport chain or, or fatty acid oxidation. HBC were incubated with apoptotic neutrophils in a 1:5 ratio and after 50 min of efferocytosis cells were collected, immunostained for CD14 and the percentage of CD14/CFSE double-positive cells was analyzed by flow cytometry as described above.

### Confocal microscopy

HBC were labeled with the lipid intercalating dye PKH26 (Sigma-Aldrich) according to the manufacturer’s instructions. Male or female macrophages were incubated with their autologous apoptotic neutrophils stained with CFSE and performed efferocytosis assay, as previously described. Then, cells were washed, fixed with paraformaldehyde 4%, and stained with DAPI. Cells were visualized and microphotograped with a confocal fluorescence Zeiss-LSM980 microscopy. Photographs were analyzed with Fiji sofware.

Negative controls were set with each fluorescent dye separately and images displayed were at 60x.

### RT-qPCR

Villi gene expression of cytokines [Interleukin (*IL*) -1β, -6, -10; Indoleamine 2,3-dioxygenase (*IDO*), Transforming growth factor (*TGF-*) β1, tumor necrosis factor (*TNF*) -α], toll-like 4 receptor (*TLR4*), prostaglandin-endoperoxide synthase 2 (*PTGS2*) and unfolded protein response (UPR) genes (*IRE-1α*, *PERK*, *ATF6α* and *ATF4*) was determined by RT-qPCR as previously described [37]. Briefly, total RNA was isolated following manufacturer recommendations with Trizol reagent (Life Technologies, Grand Island, NY, USA), cDNAs generated from 1 ug of RNA using a MMLV reverse transcriptase, RNAsin RNAse inhibitor and oligodT kit (Promega Corporation, Madison, WI, USA). Samples were stored at -20 ºC for batch analysis.

RT-qPCR master mix (Biodynamics, Buenos Aires, Argentina) was used according to the manufacturer`s recommendation. Real-time PCR was performed on a Bio-Rad iQ5 Real-time PCR system. The relative gene expression levels were determined using the threshold cycle (CT) method (2^-dCT method) with reference to two housekeeping genes that did not show sex-influence, Glyceraldehyde-3-phosphate dehydrogenase (GAPDH) and beta-2 microglobulin (B2-M). The primer sequences are described in Table 1.

**Table 1:** Primer sequences with their melting temperature

Gene	Primer Forward	Primer reverse	Tm (ºC)
ATF4	5’ GTCAGTCCCTCCAACAACAGC 3’	5’ AGAGATCACAAGTGTCATCCAACG 3’	60
ATF6α	5’ GCTGCCCTCTCAGAAAACGA 3’	5’ CCTGTTCCAACATGCTCATAGG 3’	60
B2-M	5’ AAGCAGCATCATGGAGGTTTG 3’	5’ GAGCTACCTGTGGAGCAACC 3’	58
GAPDH	5’ ATCACCATCTTCCAGGAGCGA 3’	5’ GGTTCACACCCATGACGAAC 3’	60
IDO-1	5’ AGGCCCTGATTATGAGAACAT 3’	5’ TTTCCAACAGCGCCTTTAGC 3’	60
IL-1β	5’ GGATATGGAGCAACAAGTGGTGT 3’	5’ CACGCAGGACAGGTACAGATT 3’	60
IL-6	5’ TTCGGTACATCCTCGAGGC 3’	5’ TCACCAGGCAAGTCTCCTA 3’	60
IL-10	5’ CATCAAGGCGCATGTGAACT 3’	5’ CACAGGGAAGAAATCGATGACAG 3’	60
IRE-1α	5’ GGGTCTGAGGAAGGTGATGC 3’	5’ AACATACAGAGTGGGCGTCAG 3’	60
PERK	5’ AGACGATGAGACAGAGTTGCG 3’	5’ TTGCTAAGGCTGGATGACACC 3’	58
PTGS2	5’ TGGATGCTTCGTTAATTTGTTC 3’	5’ ACCCACAGTGCTTGACAC 3’	60
TGF-β1	5’ GGACACCAACTATTGCTTCAG 3’	5’ CCAGGCTCCAAATGTAGGG 3’	56
TNF-α	5’ GCCTCTTCTCCTTCCTGATCG 3’	5’ CAGCTTGAGGGTTTGCTACA 3’	60
TRL4	5’ ATCCCCTGAGGCATTTAGGC 3’	5’ TTGTCTGGATTTCACACCTGG 3’	58

### Bioinformatic studies

#### Samples classification

Expression matrix was downloaded from NCBI GEO (accession GSE30595 [46]) using the GenePattern platform [47]. We selected samples which underwent cannonical differentiation towards M1 macrophages (GM-CSF/LPS/IFN). Consequently, nine samples were selected for subsequent enrichment analysis, differential expression, and gene contrasts.

To determine the sex of each selected sample, the expression of genes associated with the Y chromosome (UTY, TMSB4Y, EIF1AY, USP9Y, RBMY1A1) were detected by the microarray and clustering by PCA on R [48].

#### Differential gene expression and enrichment analysis

We studied differentially expressed genes (DEG) and contrasted between female vs. male samples in each activation profile using the limma package in R [49] (Log_2_FC > 0.58, adjusted P-value < 0.05). Subsequently, Gene set enrichment analysis (GSEA) dot plots was obtained with gseapy package [50] in Python.

### Statistical analysis

The significance of the results was analyzed by Wilcoxon matched-pairs or Mann-Whitney U-test for two nonparametric samples. When multiple comparisons were necessary, One- or two-way ANOVA test followed by Holm-Sidak or Tukey’s multiple comparison tests were used. Differences between groups were considered significant at *P* < 0.05 using the GraphPad Prism9 software (GraphPad, San Diego, CA, USA).

## Results

### Female Hofbauer cells are more prone to an alternative-like profile

**Fig. 1:**
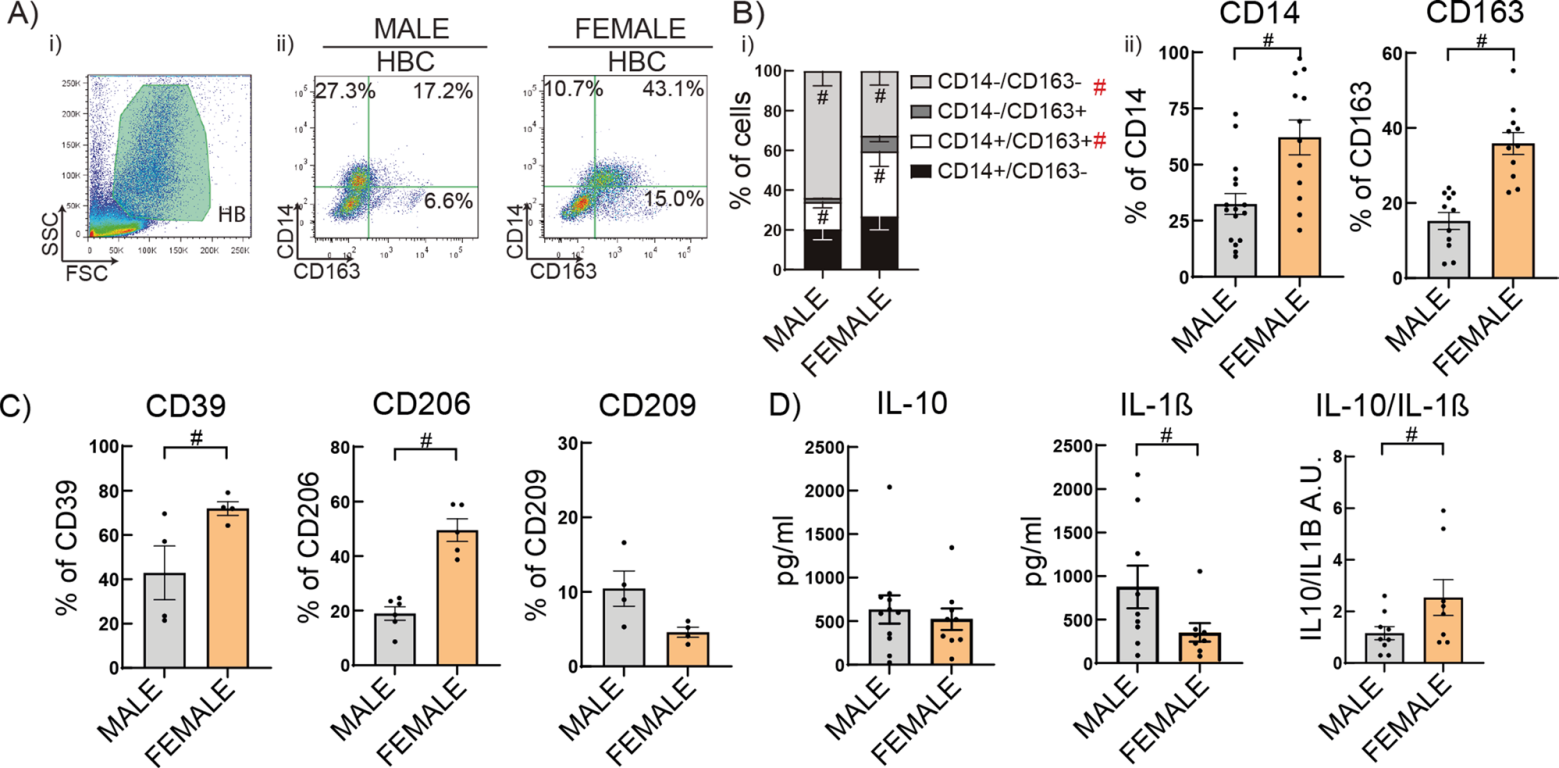
Female Hofbauer cells are more prone to an alternative-like profile. HBC were isolated from 31 placentas (16 male and 15 female newborn) by enzymatic digestion and cultured with RPMI 2% FCS ON. Cells were collected and analyzed by flow cytometry. **A**) Representative dot plots of (i) HBC gate and (ii) CD14-CD163% of positive cells. **B**) CD14 and CD163 proportion and **C**) CD39, CD206, CD209% of positive cells were shown. **D**) IL-10 and IL-1β secretion (pg/ml) were measured by ELISA in HBC supernatants. Results are expressed as Mean ± SEM. Statistically significant differences by placental sex are represented as in B) # (red) *P* < 0.05. Two-way ANOVA, Tukey’s multiple comparisons test [Interaction: F(3) = 8.628, P-value < 0.0001; Sex: F(1) = 5.99e-008, P-value = 0.998; Cell population: F(3) = 20.64, *P* < 0.0001]. B-D) # *P* < 0.05 (Mann-Whitney test) when only 2 conditions were analyzed. Each dot represents an independent experiment

HBC cells from female term placentas (FEMALE) showed increased co-expression of CD14 and CD163/SCARI1 (scavenger receptor cysteine-rich SRCR) compared to male HBC cells (MALE) (Fig. 1A-B). Additionally, Female HBC exhibited higher expression of antiinflammatory surface markers such as CD39/ENTPD1 (ectonucleoside triphosphate diphosphohydrolase 1) and CD206/MRC1 (mannose receptor C-type 1) (*P* < 0.05), but not CD209/DC-SIGN (C-type lectin) (Fig. 1C). This profile was accompanied by a decrease in IL-1β secretion without changing IL-10 secretion and a higher IL-10/IL-1β ratio in female compared to male HBC (Fig. 1D) (*P* < 0.05). Interestingly, these sex-specific differences in the phenotypical profile of HBC have a correspondence in monocyte-derived M1 macrophages from male volunteers compared to female, as acquired using public data (GSE30595) displayed in Supplementary Fig. 1.

### Male HBC display a preferential glycolytic metabolism whereas female HBC rely on fatty acid uptake and accumulation of lipid droplets

**Fig. 2:**
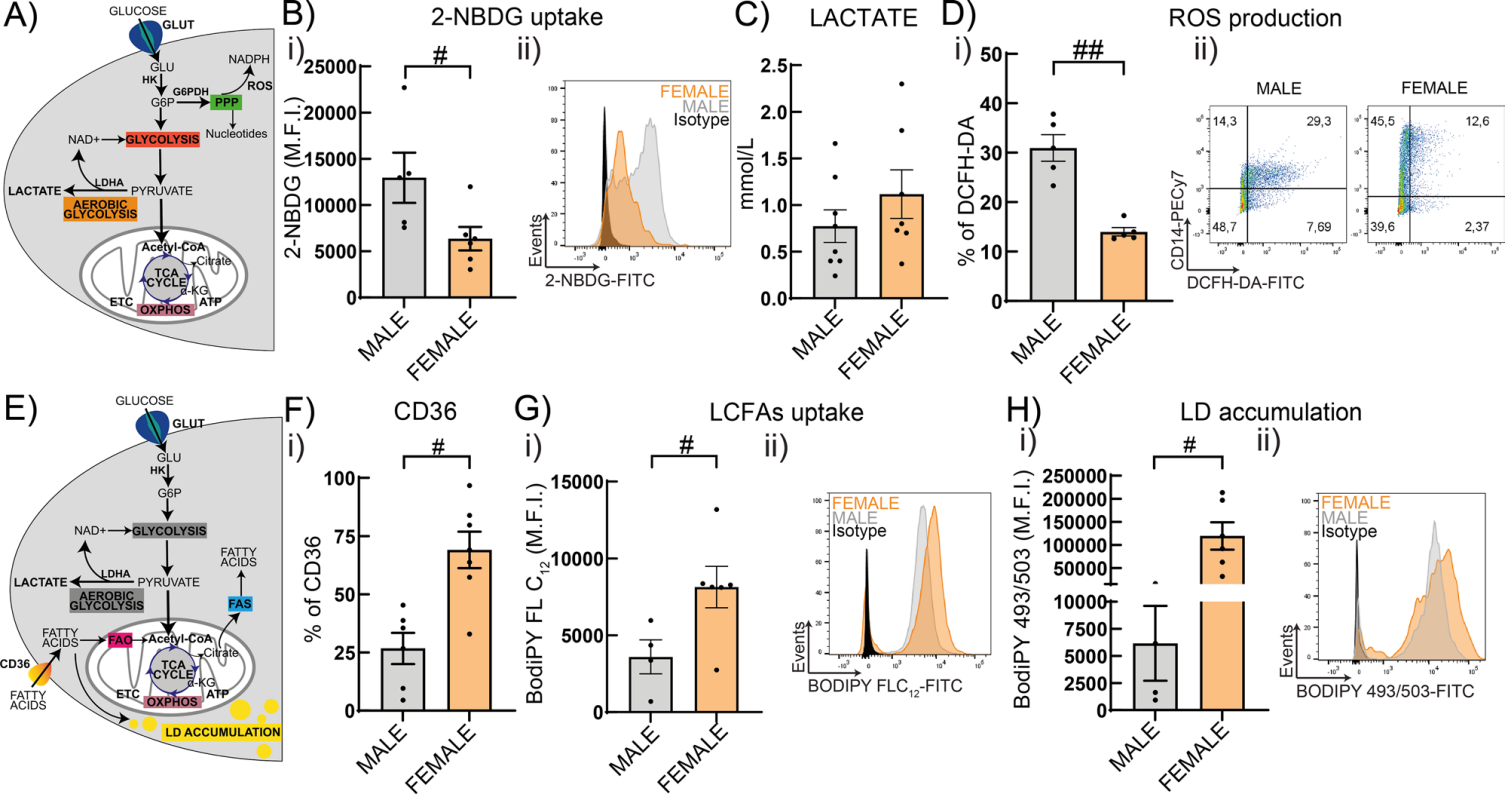
Differential metabolic preferences in male and female HBC. **A**) and **E**) Simplified schematic overview of glucose and lipid metabolism, respectively. HBC cells were cultured with RPMI 2% FCS ON and collected to perform metabolic assays to study **B**) the glucose analogue 2-NBDG uptake, **D**) ROS production, **F**) CD36 expression, **G**) LCFAs uptake and **H**) Lipid Droplets (LD) accumulation by flow cytometry. Supernatants were used to study **C**) Lactate secretion with a colorimetric assay. Results are expressed as Mean ± SEM and in ii) representative histogram or dot plots are shown. Statistically significant differences between sex are represented as # *P* < 0.05 (Mann-Whitney test). Each dot represents an independent HBC sample. Abbreviations: Glucose (GLU), hexokinase (HK), glucose-6-phospate (G6P), G6P dehydrogenase (G6PDH), pentose phosphate pathway (PPP), reactive oxygen species (ROS), Lactate dehydrogenase A (LDHA), Tri-carboxylic acid (TCA), electron transport chain (ETC), oxidative phosphorylation (OXPHOS), fatty acid oxidation (FAO), fatty acid synthesis (FAS)

On the basis that the immunometabolic reprograming of macrophages underlies the phenotypic profile acquired [32, 34, 51, 52], we next explored whether the sex-associated phenotype of HBC was related to a differential metabolic profile. Male HBC showed higher uptake of the glucose analogue, 2-NBDG (Fig. 2B), higher production of total reactive oxygen species (ROS) (*P* < 0.01) and similar levels of lactate secretion compared to female HBC (Fig. 2C-D). On the other hand, Female HBC displayed higher long chain fatty acids (LCFA) incorporation (BodiPY FLC_12_) (Fig. 2G) accompanied by higher production of CD36 and lipid droplets accumulation (BodiPY 493/503) than male HBC (Fig. 2F, H) (*P* < 0.05). These results are in line with sex-associated metabolic pathways accompanying phenotypic differential profiles.

### Higher efferocytic capacity and differential efferocytosis fueling in female HBC

**Fig. 3:**
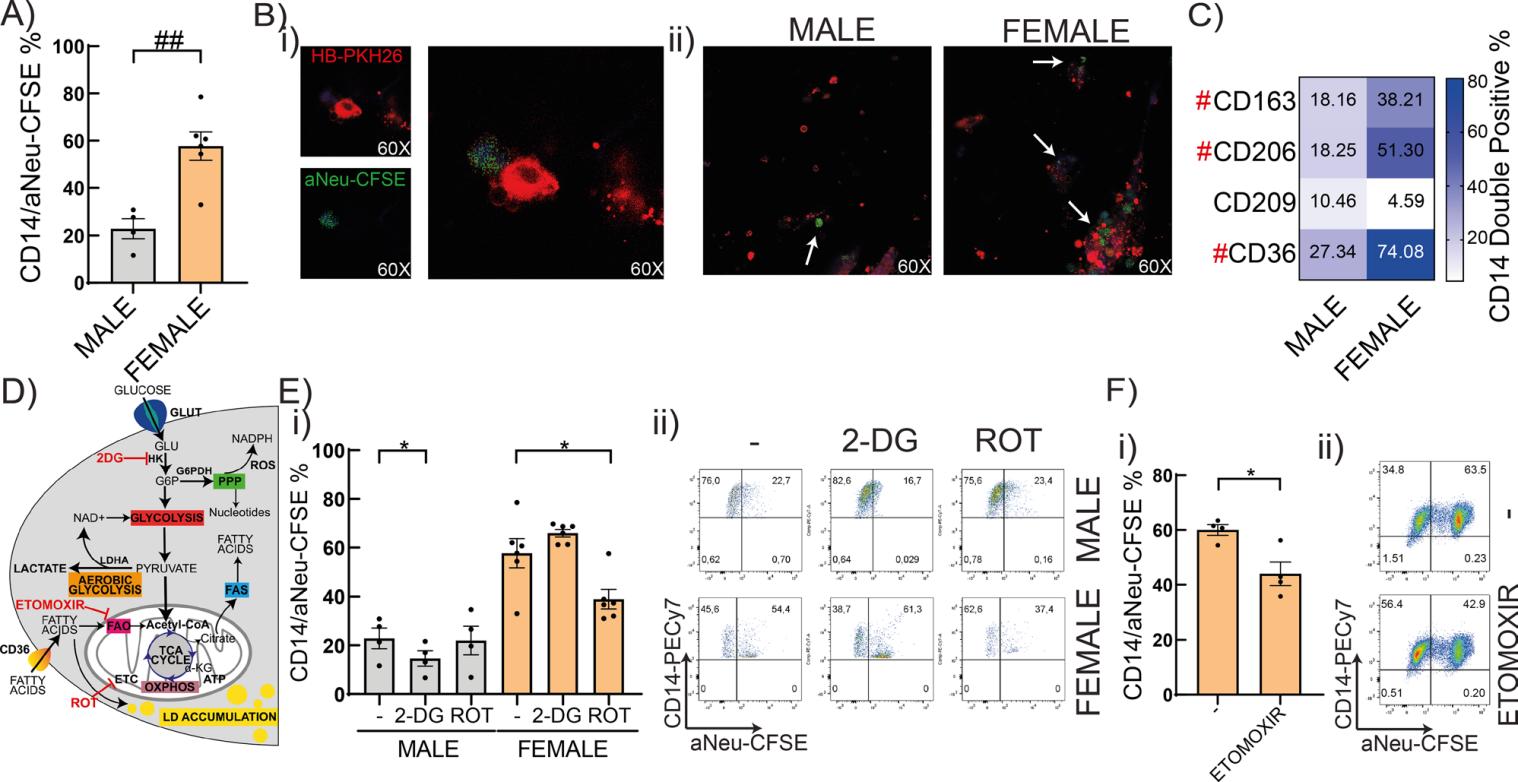
Sex-associated metabolic pathways in efferocytosis of HBC. **A**) Male or female HBC were challenged with maternal apoptotic neutrophils (aNeu), stained with CFSE, in a 1:5 ratio during 1 h. Then, they were washed and incubated with CD14 antibody. Double positive cells were analyzed by flow cytometry. **B**) Representative microphotographs of efferocytosis process (i) and MALE and FEMALE efferocytosis (ii) were taken by Zeiss LSM980 confocal microscope of 3 independent experiment each. White arrows show apoptotic bodies (green) in HBC-PKH26 (red). **C**) Phagocytic receptors expression (CD163, CD206, CD209 and CD36) were analyzed by flow cytometry. **D**) Simplified schematic overview of metabolic pathways and pharmacological inhibitors. **E**), **F**) HBC cells were cultured with metabolic inhibitors 2-DG (10 mM), ROT (100 nM) or ETOMOXIR (10 µM), stimulated with apopototic neutrophils as in (A) and analyzed by flow cytometry. Results are expressed as Mean ± SEM. Statistically significant differences: A, C) between HBC sex are shown as # *P* < 0.05, ## *P* < 0.01 (Mann-Whitney test). E) * *P* < 0.05, ** *P* < 0.01. Two-way ANOVA, Holm-Sidak’s multiple comparisons test. [Interaction: F(2) = 11.62, P-value = 0.0006; Sex: F(1) = 70.85, P-value < 0.0001; Treatment: F(1) = 6.157, *P* = 0.0095]. F) * *P* < 0.05 (basal vs. ETOMOXIR, Wilcoxon matched-pairs signed rank test). Each dot represents an independent HBC sample

First, we assessed the efferocytic capacity of HBC as the ability to remove maternal apoptotic neutrophils. As shown in Fig. 3, female HBC have almost three times higher efferocytosis than male HBC as assessed by flow cytometry (Fig. 3A) (*P* < 0.01) and visualized by confocal microscopy (Fig. 3B). These results were accompanied by an increase in the expression of the efferocytosis receptors CD36, CD163 and CD206 (Fig. 3C) (*P* < 0.05), congruently with their alternative profile (Fig. 1). To explore further the metabolic preferences of male and female HBC, we studied their efferocytic capacity in the presence of different metabolic inhibitors. To assess glucose or mitochondrial dependent efferocytosis, the competitive inhibitor of glycolysis 2-deoxy-d-glucose (2-DG) or Rotenone (ROT) were added to HBC cultures before the efferocytosis assays, as depicted in Fig. 3D). The inhibitor of glucose utilization 2-DG reduced efferocytosis up to 35% only in male HBC (*P* < 0.05), with no changes in female HBC (Fig. 3E). In contrast, the implication of mitochondrial energy for this function was more evident in female HBC, since the electron transport chain inhibitor ROT reduced up to 50% the efferocytosis in female (*P* < 0.01) but not in male HBC (Fig. 3E). Finally, female HBC were cultured with Etomoxir, a CPT1-specific inhibitor to prevent the transport of fatty acids into the mitochondrial matrix for further metabolization. As shown in Fig. 3F, female HBC obtain energy for efferocytosis, at least partially, through fatty acid oxidation (Fig. 3E) (*P* < 0.05).

### Differential activation of HBC metabolism by LPS

**Fig. 4:**
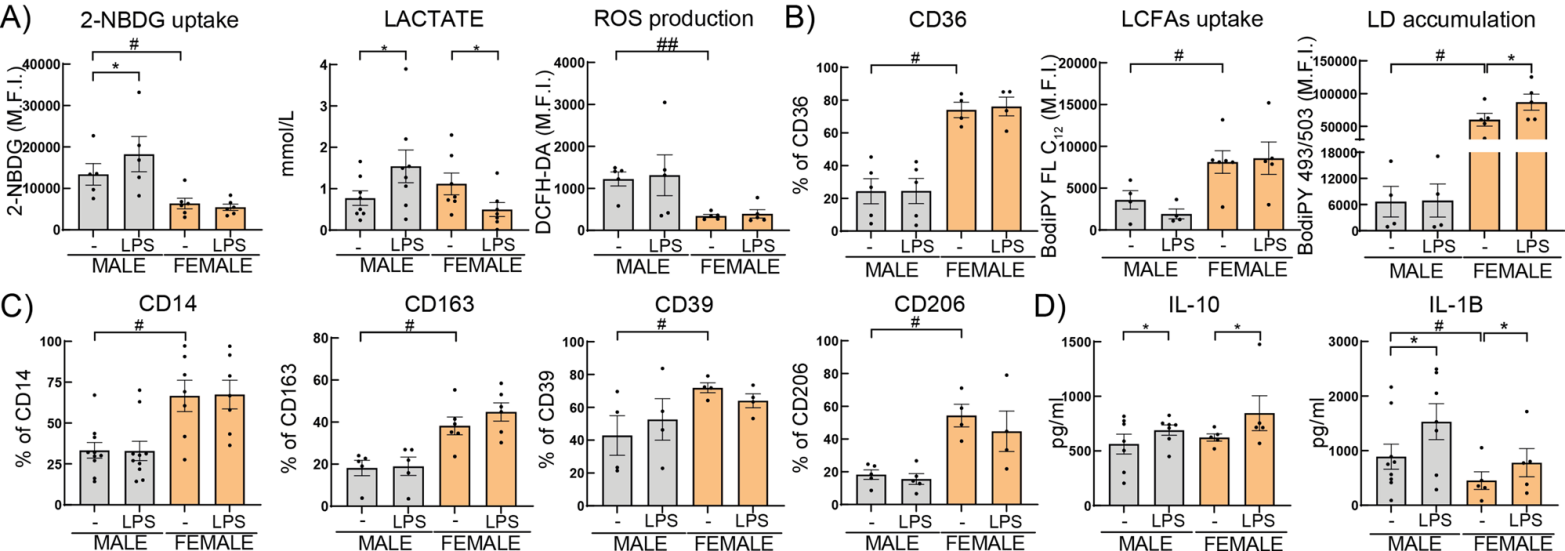
HBC immunometabolic profile modulated by LPS. Male or female HBC were cultured in RPMI 2% FCS ON. **A**) glucose uptake and ROS production, **B**) CD36, LCFAs uptake, lipid droplets (LD) accumulation and **C**) CD14, CD163, CD206 and CD39, were measured by flow cytometry. In the supernatants **A**) Lactate or **D**) IL-10 and IL-1Β secretion were quantified by enzymatic assay or ELISA, respectively. Results are expressed as Mean ± SEM. Statistically significant differences by the stimuli within each sex are represented as * *P* < 0.05 (Wilcoxon matched-pairs signed rank test) and those parameters that were detected as significantly different between basal male and female HBC in Figs. 1 and 2, are here marked with #. Each dot represents an independent HBC sample

Next, we studied the metabolic rewiring of male or female HBC when challenged ex vivo with a classical proinflammatory stimulus as lipopolysaccharide (LPS). An enhanced glycolytic metabolism with higher glucose analogue uptake and lactate secretion was observed in male HBC (Fig. 4A) (*P* < 0.05). LPS did not affect free fatty acid uptake, lipid droplet accumulation and antiinflammatory surface marker expression (Fig. 4B, C). On the other hand, female HBC cultured with LPS showed lower secretion of lactate (*P* < 0.05) without changes in glucose uptake or ROS production (Fig. 4A). Nevertheless, an increment in lipid droplets (LD) accumulation (Fig. 4B) (*P* < 0.05) and a trend to increase in CD163 was observed (Fig. 4C). Both male and female HBC secreted more IL-1β and IL-10 upon LPS challenge (Fig. 4D) (*P* < 0.05). These results indicate that male HBC’s metabolism challenged by LPS responded similarly to myeloid macrophage, increasing glycolytic metabolism and secretion of proinflammatory mediators, as a classic activation profile. In contrast, female HBC challenged by LPS did not rewire their metabolism to glycolysis, but rather maintained that of an alternative activation profile.

### IRE-1 regulates differently the immunometabolic profile of HBC

**Fig. 5:**
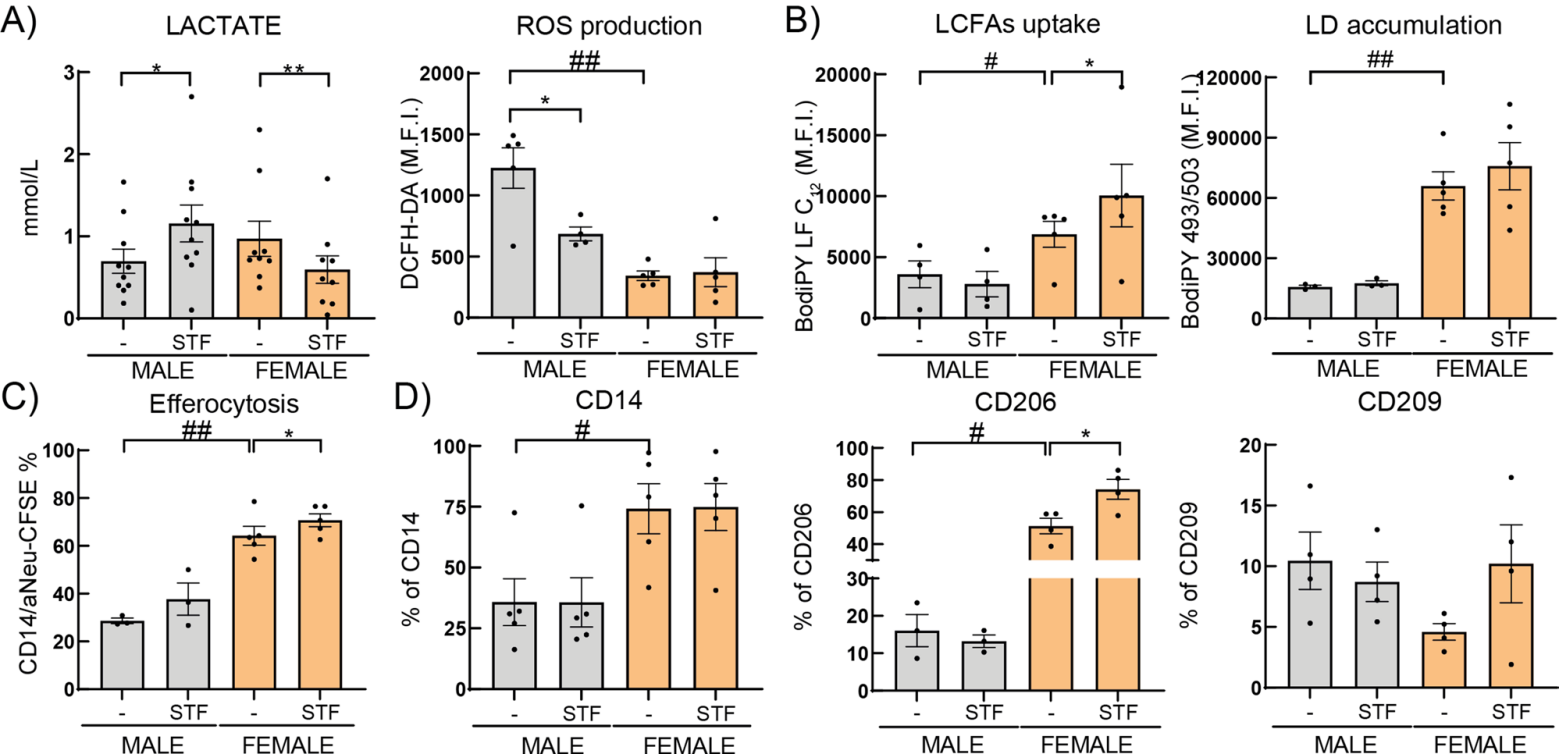
Pharmacological inhibition of IRE-1 and its association to HBC metabolic profile. HBC were cultured without or with 10μg/ml IRE1α inhibitor, STF-083010 (STF) in RPMI 2% FCS ON. **A**) ROS production, **B**) LCFA uptake and LD accumulation, **C**) Efferocytosis and **D**) CD14, CD206 and CD209 positive cells were evaluated by flow cytometry. In the supernatant **A**) Lactate was quantified by enzymatic assay. Results are expressed as Mean ± SEM. Statistically significant differences by the stimuli within each sex are represented as * *P* < 0.05 (Wilcoxon matched-pairs signed rank test) and those parameters that were detected as significantly different between basal male and female HBC in Figs. 1, 2 and 3, are here marked with #. Each dot represents an independent HBC sample

Components of the unfolded protein response (UPR) regulate various processes, from lipid and cholesterol metabolism and energy homeostasis to inflammation and cell differentiation [53, 54]. One of the sensors of the UPR, the inositol-requiring protein 1 (IRE-1), is widely expressed by macrophages and activated by endoplasmic reticulum stress [54–56]. As a potential cellular mechanism for the sex-associated differential immunometabolic profiles, we studied the involvement of IRE-1 in HBC. Since IRE-1 associates with metabolic pathways as glucose fluctuation, lipid and/or cholesterol biosynthesis and regulates cytokines associated to secretion in macrophages [54, 56, 57], we performed experiments with its pharmacological inhibitor STF-083010 (STF). Male HBC treated with STF increased lactate secretion and decreased ROS production (*P* < 0.05) without changing lipid metabolism, efferocytosis or scavenger receptor expression (Fig. 5A-D). In contrast, female HBC cultured with STF decreased lactate secretion (*P* < 0.01) without changing ROS production, but it increased LCFA uptake (*P* < 0.05) and tended to increase LD accumulation (Fig. 5A-D). Surprisingly, STF enhanced efferocytosis, CD206 and a slight CD209 increase only in female HBC (Fig. 5A-D) (*P* < 0.05). Altogether, these results suggest that in a physiological stress response, IRE-1α is regulating the predominant M2 phenotype of female but not of male HBC.

### Contribution of the placental microenvironment to sex-associated differences of HBC

**Fig. 6:**
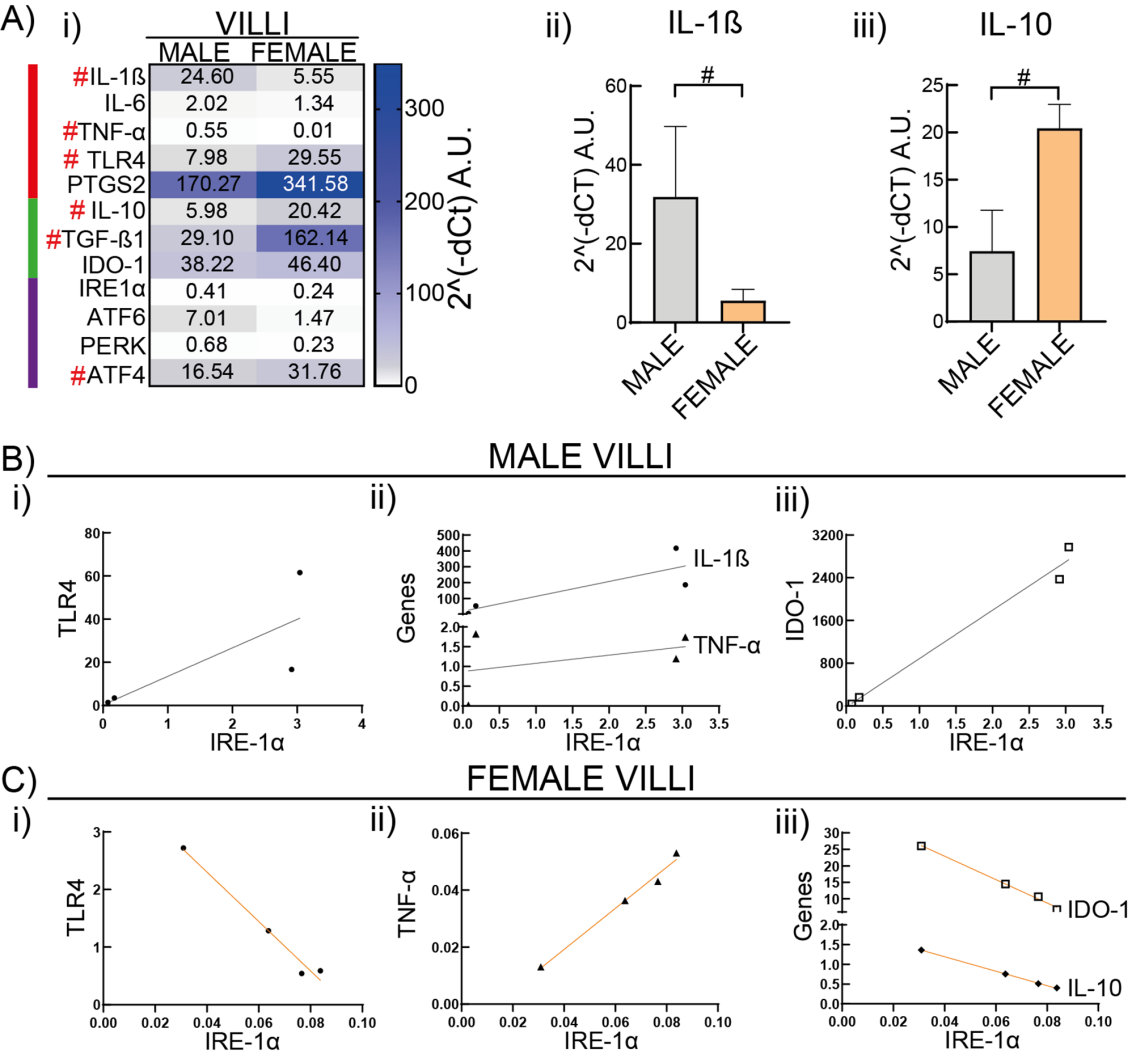
Sex associated transcriptionally differences in the placental microenvironment. Placentas (from 4 male and 4 female births) were processed to study gene expression in villi explants by RT-qPCR. **A**) Male or female gene expression is shown as their Mean 2^(-dCt). Statistically significant differences between sex are shown by # *P* < 0.05 (Mann-Whitney test). Red, green and purple lines mark pro, antiinflammatory and UPR genes, respectively. **B**) Male or **C**) Female gene correlations were evaluated by Pearson’s r. The r value was positive (blue) or negative (red) when the gene correlation was positive or negative, respectively. All correlations that are shown have P-values < 0.05

To understand if the evidence of sex-specific diversity in the HBC is intrinsic to the cell or it also extends to the surrounding environment of the placental villi, we evaluated the expression of certain selected genes contributing to pro-, antiinflammatory responses or endoplasmic reticulum stress in placental explants. Villi gene expression was normalized against two housekeeping genes that did not show sex-influence, Glyceraldehyde-3-phosphate dehydrogenase (GAPDH) and beta-2 microglobulin (B2-M). In female villi, as expected according to isolated cells data, there was lower expression of proinflammatory genes such as IL-1β and TNF-α, but higher expression of antiinflammatory genes, IL-10 and TGF-β1 compared to male villi (*P* < 0.05). Notably, female villi expressed higher expression of TLR4 and ATF4 a UPR gene (Fig. 6A) (*P* < 0.05). Interestingly, IRE-1α is the only UPR sensor expressed in all samples, whereas PERK and ATF6α were expressed in a few samples independently of the placenta sex (Supplementary Fig. 2). Moreover, when plotting the expression of IRE-1α vs. different genes in male and female villi, an inverse correlation was observed for TLR4 and IDO-1 whereas differential steepness was obtained for TNF-α. Taken together, these results support the proposal that villi inherent factors associated to the inflammatory and reticulum stress responses have a role in the sex-specific features described in female and male HBC, with female villi genes contributing mostly to antiinflammatory profiles than male villi.

## Discussion

Here, we present evidence of sex-specific differential immunometabolic, functional and phenotypical profiles of HBC in human term placentas. Results also point to the preferential metabolic pathways involved in the efferocytic function of HBC. We propose that placental macrophages’ function depends on the preferentially selected metabolic pathways, independently of cellular access to nutrients. Our results support that female HBC have a prominent M2 phenotype associated to lipid metabolism; instead, male HBC present a weaker M2 phenotype associated to glycolytic pathways. These conclusions are based on the following observations. First, a higher expression of CD14 and CD163 was confirmed in female HBC than male HBC [4, 5], along with greater expression of CD39, CD206 as well as lower secretion of IL-1β. Second, male HBC displayed higher glucose analogue uptake and ROS production than female HBC, whereas female HBC presented higher fatty acid uptake and accumulation of lipid droplets, accompanied by higher production of the scavenger receptor CD36. Third, female HBC displayed almost three times more efferocytosis than male HBC, as well as enhanced expression of CD36, CD163 and CD206. Noteworthy, energy for efferocytosis was obtained, at least partially, from fatty acid oxidation in female but from glycolysis in male HBC, as corroborated with pharmacologic inhibitors. Fourth, the predominant metabolic pathways in male HBC were reinforced upon LPS challenge, since increased glucose uptake and lactate secretion without changing lipid mobilization and accumulation was observed; in contrast, female HBC presented lower secretion of lactate and higher lipid droplet accumulation without changing glucose or fatty acid uptake. Fifth, IRE-1α inhibitor favored lactate secretion but decreased ROS production without changing efferocytosis or the expression of receptors associated to this process in male HBC. In female HBC, IRE-1α inhibition reduced lactate secretion, increased fatty acid uptake and strengthened even more the efferocytic capacity in female HBC as well as increased CD206 expression. Sixth, sex-differences studied in isolated HBC were observed in term placenta villi as well: female villi contributed less to a proinflammatory profile while IRE-1α expression correlated differently with pro- and antiinflammatory genes.

Our data on HBC immunometabolic profiles according to the sex of the placenta fits in the epidemiologically registered sex-associated differences of maternal and fetal outcomes related to the function of the feto-placental unit. HBC are originated in the placenta and share genetic information with the fetus. Differences among these cells are not only genetical [5, 22, 58] but also environmental [12, 13] and depending on gonadal hormones [1, 12, 59, 60]. Sexual hormones are known to modulate macrophage classical innate function in adult males and females [61–63]. However, hormonal independence in the sex-specific metabolic preferences of macrophages was recently described in adrenal gland macrophages where ovaries-derived hormones appeared unlikely regulators of sex dimorphism [64]. In this work, we opted to focus on factors inherent to the female or male villi, which include the hormonal environment, rather than on the hormones alone. Our results indicate that female villi contributed to antiinflammatory profiles and strongly support a role for inflammatory and endoplasmic reticulum stress mediators in the effect. This was not as evident in the males pointing out that HBC immunometabolic differential profiles rely on a more complex regulatory network that includes inflammatory, UPR and ER stress signals. Moreover, these differential sex-associated pathways of HBC accompanying their phenotypic and functional profiles seem to occur in the villi environment before cell isolation, since the ex vivo assays were carried out in the same metabolic experimental settings.

Efferocytosis, macropinocytosis and phagocytosis together with diverse scavenger receptors (including CD36, CD163, CD206) are involved in lipid uptake by macrophages [65]. The fact that female HBC had more efferocytosis accompanied by a stronger surface expression of CD36, CD163 and CD206 [28, 66] and that rotenone and etomoxir decreased efferocytosis strongly support the preferential use of fatty acid oxidation to get energy for this process. In contrast, male HBC, under the same nutrient supply, preferred the faster glycolytic metabolism to perform functions as efferocytosis in vitro (Figs. 2 and 3). This result adds new metabolic evidence to explain the observed higher susceptibility of male fetuses to various insults that alter cellular metabolism [3].

Interestingly, female placental macrophages displayed a more prominent M2 phenotype than male macrophages whereas they better controlled the inflammatory response to LPS. It is conceivable that these mechanisms might underlie the impact of their response against environmental and infectious insults as shown in placental [12] and immune tissues [62]. These results are in line with Pantazi et al. [28] who reported a stronger response of female term HBC to LPS than male HBC. Regarding the differences seen in TLR4 and the response to LPS it is worthy to note that TLR4 is expressed by HBC from early stages of pregnancy until term [25] but its role in the immunometabolism of HBC regarding sex has not been assessed. Remarkably, gene expression relationships in villi indicate that TLR4 is highly expressed and correlates negatively with TNF-α but positively with IDO-1 and IL-10 in female placentas (Fig. Suppl 2). On the other hand, male HBC cultured with LPS showed a reinforced glycolytic metabolism favoring the aerobic glycolysis instead of the oxidative pentose phosphate pathway; and it was accompanied by IL-1β and IL-10 secretion (Fig. 4). Similarly, TLR4 correlates positively with IL-6 and TGF-β1 on male villi (Supplementary Fig. 3). This preferential glycolytic metabolic pathway associated to the higher number of proteins related to cholesterol (steroids) and alcohol biosynthesis [28], it may be a link to explain why HBCs are activated in a weaker M2 phenotype than females HBC.

We found a strong association of the IRE-1 pathway to the phenotypic, functional and metabolic profile of isolated HBC and villus tissue as well (Figs. 5 and 6), being more significant in female samples. This may suggest that, given the demanding functions that placental macrophages need to fulfill [21, 67], depending on their metabolism they react differently to the physiological stress. This differential bias in how IRE-1α acts in male and female HBC should be further explored to understand how it is involved in the immunometabolism of HBC and whether it affects pregnancy outcome.

Immunometabolism and pregnancy is a novel area of research with only a few papers about immunometabolic profiles of decidual macrophages [33, 35, 67] and a recent report from our group studying maternal monocytes’ immunometabolism at 16–20 weeks of pregnancy [34]. To our knowledge, this is the first report on HBC immunometabolism and, especially, the first taking the particular approach of sex-associated differences. Sex-associated diversity in Hofbauer cell immunometabolism may contribute to the understanding of sex-specific susceptibility to infections during pregnancy and other pregnancy complications. Investigating these differences may offer insights into the pathogenesis of conditions such as preeclampsia and intrauterine growth restriction, opening avenues for targeted interventions.

## Electronic supplementary material

Below is the link to the electronic supplementary material.

Supplementary Material 1

## Data Availability

No datasets were generated or analysed during the current study.

## Abbreviations

2-DG: 2-deoxy-d-glucose
2-NBDG: 2-Deoxy-2-[(7-nitro-2,1,3-benzoxadiazol-4-yl)amino]-D-glucose
aNEU: Apoptotic neutrophils
ATF-: Activating transcription factor
APC: Allophycocyanin
B2M: Beta-2 microglobulin
BMI: Body Mass Index
BODIPY: 4,4-difluoro‐3a,4a‐diaza‐s‐indacene
CFSE: Carboxyfluorescein succinimidyl ester
DCFH-DA: 2’,7’-Dichlorodihydrofluoresceindiacetate
DEG: differentially expressed genes
DMEM: Dulbecco’s Modified Eagle Medium
EDTA: Ethylenediamine tetraacetic acid
EIF1AY: Eukaryotic Translation Initiation Factor 1 A Y-Linked
ELISA: Enzyme-Linked ImmunoSorbent Assay
ER: Endoplasmic reticulum
ETC: electron transport chain
FCS: Fetal calf serum
FITC: Fluorescein Isothiocyanate
FAF-BSA: fatty acid free bovine serum albumin
FAO: fatty acid oxidation
FAS: fatty acid synthesis
G6P: glucose-6-phospate
G6PDH: G6P dehydrogenase
GAPDH: Glyceraldehyde-3-phosphate dehydrogenase
GM-CSF: Granulocyte macrophage colony-stimulating factor
GSEA: Gene set enrichment analysis
GLU: Glucose
HBC: Hofbauer cells
HK: hexokinase
IDO: Indoleamine 2,3-dioxygenase
IFN: Interferons
IRE-1: Inositol-requiring enzyme-1
LCFA: Long chain fatty acid
LD: Lipid droplets
LDHA: Lactate dehydrogenase A
LPS: Lipopolysaccharide
MFI: Mean fluorescence intensity
OXPHOS: oxidative phosphorylation
PE: Phycoerytrin
PECy7: Phycoerytrin-Cyanine7
PERK: Protein Kinase R-like ER Kinase
PPP: Pentose phosphate pathway
RBMY1A1: RNA binding motif protein Y-linked family 1 member A1
ROS: Reactive oxygen species
ROT: Rotenone
RPMI: Roswell Park Memorial Institute 1640
STF: STF-083010
TCA: Tri-carboxylic acid cycle
TGF: Transforming growth factor
TLR: Toll like receptor
TMSB4Y: Thymosin Beta 4 Y-Linked
TNF: Tumor necrosis factor
UPR: Unfolded protein response
USP9Y: Ubiquitin Specific Peptidase 9 Y-Linked
UTY: Ubiquitously transcribed tetratricopeptide repeat containing, Y-linked
