# Supplementary material for: Sex-specific phenotypical, functional and metabolic profiles of human term placenta macrophages
Source: Biol Sex Differ. 2024 Oct 17;15:80. doi: 10.1186/s13293-024-00652-w (PMC11484421; doi:10.1186/s13293-024-00652-w)
Supplement: Supplementary file 1 — Supplementary Material 1 [file 13293_2024_652_MOESM1_ESM.doc]

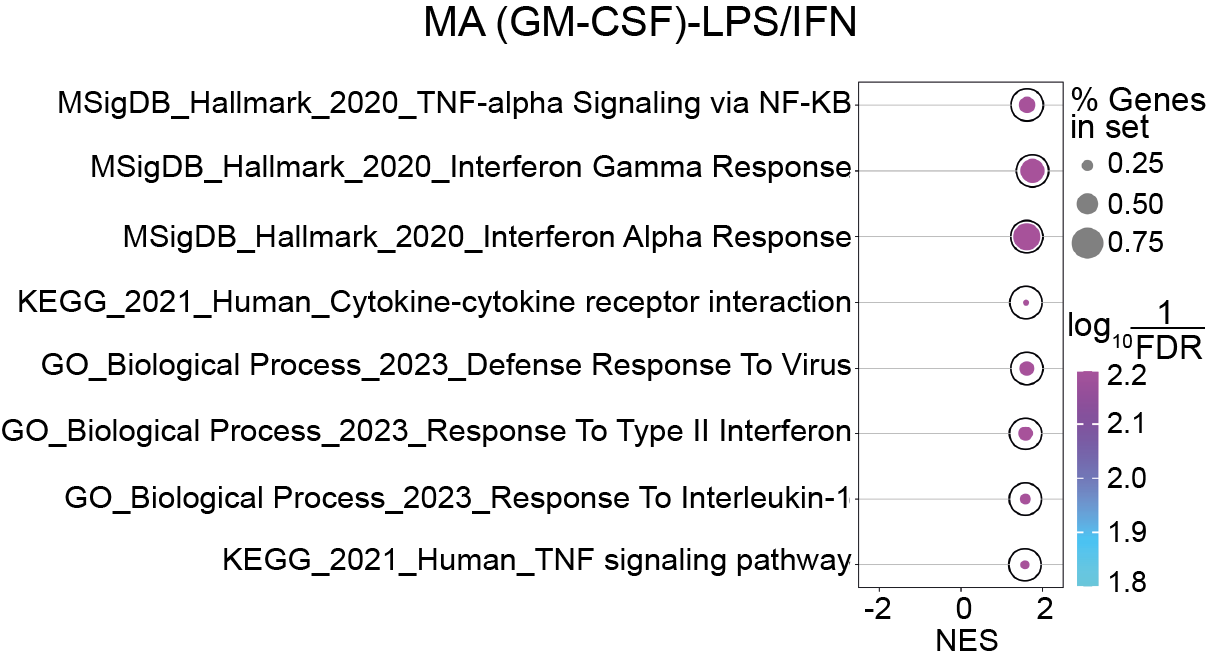


**Supplemental Fig. 1. Sex differences in macrophages derived from monocytes from healthy volunteers**. Sex-specific gene expression analysis of macrophages samples was performed using the public data provided by GenePattern platform from NCBI GEO accession GSE30595: enrichment analysis, when the diameter is bigger and stronger the purple, more genes are involved in the pathway. Colors depicted log_10_ (1/FDR) where negative or positive values are female or male samples, respectively.


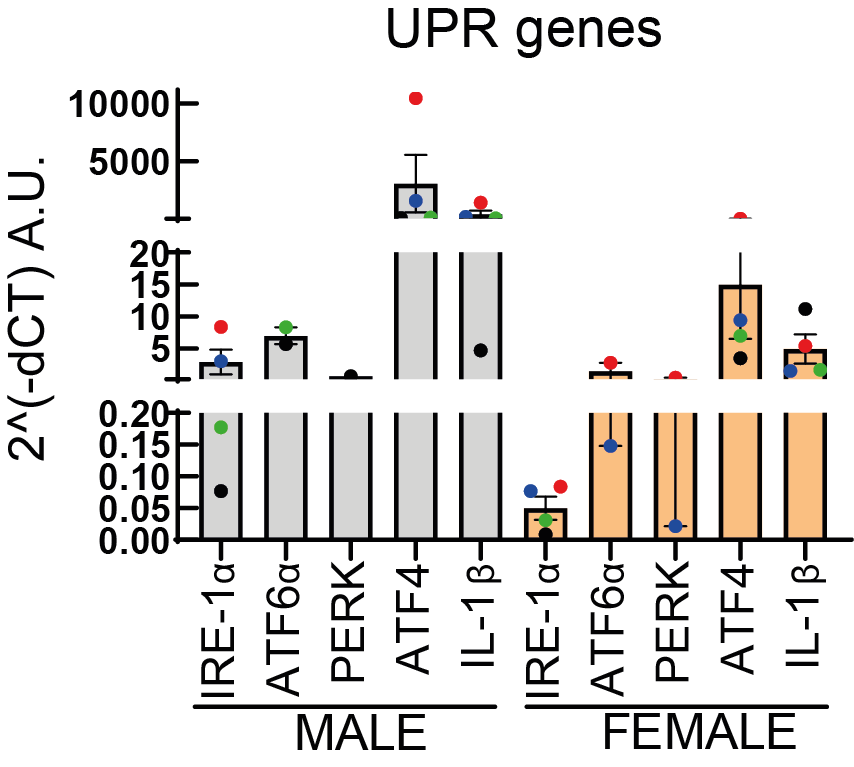


**Supplemental Fig. 2. UPR genes expression in villi explants**. 8 placentas (4 from male and 4 from female neonates) were processed to study UPR sensors, ATF4 and IL-1β gene expression by RT-qPCR. The expression is shown as Mean ± SEM of 2^(-dCt) in male or female placentas. Each color dot represents the measurement for the same placenta villi.


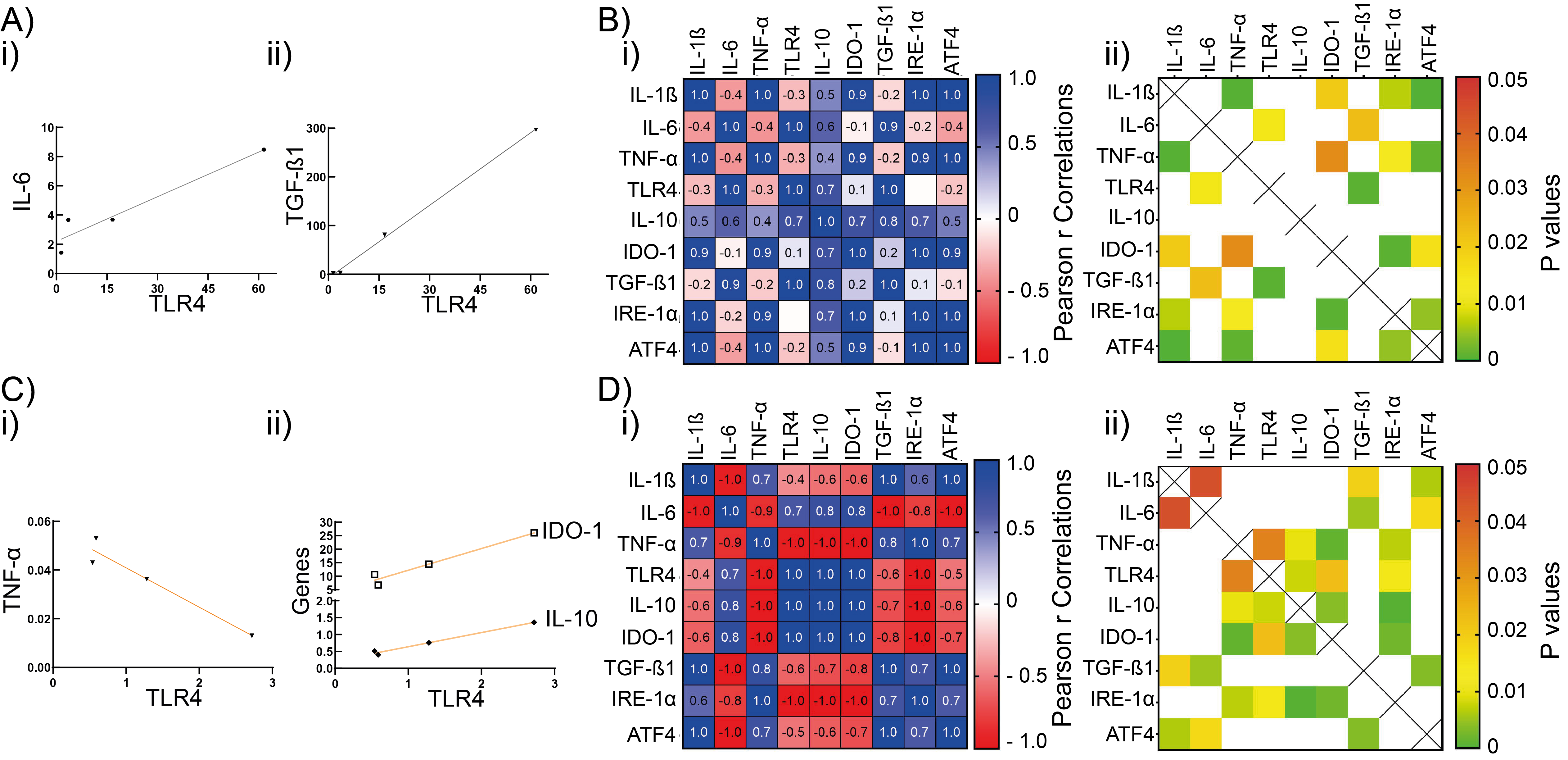


**Supplemental Fig. 3. Sex differences associated to villi expression microenvironment**. 8 placentas (4 from male and 4 from female neonates) were processed to study gene expression by RT-qPCR. A) Male or C) Female gene correlations of i) proinflammatory or ii) antiinflammatory genes correlated to TLR4 were evaluated by Pearson’s r. B, D) The r value was positive (blue) or negative (red) when the gene correlation was positive or negative, respectively. All correlations that are shown in P-values matrix of Bii) male or Dii) female villi have P < 0.05.
